# CD9 and CD81 Interactions and Their Structural Modelling in Sperm Prior to Fertilization

**DOI:** 10.3390/ijms19041236

**Published:** 2018-04-19

**Authors:** Michaela Frolikova, Pavla Manaskova-Postlerova, Jiri Cerny, Jana Jankovicova, Ondrej Simonik, Alzbeta Pohlova, Petra Secova, Jana Antalikova, Katerina Dvorakova-Hortova

**Affiliations:** 1 Group of Reproductive Biology, Institute of Biotechnology, Czech Academy of Sciences, v.v.i., BIOCEV, Prumyslova 595, 252 50 Vestec, Czech Republic; Michaela.Frolikova@ibt.cas.cz (M.F.); pavla.postlerova@ibt.cas.cz (P.M.-P.); Ondrej.Simonik@ibt.cas.cz (O.S.); Alzbeta.Pohlova@ibt.cas.cz (A.P.); 2 Department of Veterinary Sciences, Faculty of Agrobiology, Food and Natural Resources, University of Life Sciences Prague, Kamycka 129, 165 00 Prague, Czech Republic; 3 Laboratory of Structural Bioinformatics of Proteins, Institute of Biotechnology Czech Academy of Sciences, v.v.i., BIOCEV, Prumyslova 595, 252 50 Vestec, Czech Republic; Jiri.Cerny@ibt.cas.cz; 4 Laboratory of Reproductive Physiology, Institute of Animal Biochemistry and Genetics Centre of Biosciences Slovak Academy of Sciences, Dubravska Cesta 9, 845 05 Bratislava, Slovakia; jana.jankovicova@savba.sk (J.J.); petra.secova@savba.sk (P.S.); Jana.Antalikova@savba.sk (J.A.); 5 Department of Biochemistry, Faculty of Science, Charles University, Hlavova 2030/8, 128 43 Prague, Czech Republic; 6 Department of Zoology, Faculty of Science, Charles University, Vinicna 7, 128 44 Prague, Czech Republic

**Keywords:** CD9, CD81, tetraspanin network, sperm, membrane fusion, capacitation, acrosome reaction, fertilization, structural modelling, mouse, human

## Abstract

Proteins CD9 and CD81 are members of the tetraspanin superfamily and were detected in mammalian sperm, where they are suspected to form an active tetraspanin web and to participate in sperm–egg membrane fusion. The importance of these two proteins during the early stages of fertilization is supported by the complete sterility of CD9/CD81 double null female mice. In this study, the putative mechanism of CD9/CD81 involvement in tetraspanin web formation in sperm and its activity prior to fertilization was addressed. Confocal microscopy and colocalization assay was used to determine a mutual CD9/CD81 localization visualised in detail by super-resolution microscopy, and their interaction was address by co-immunoprecipitation. The species-specific traits in CD9 and CD81 distribution during sperm maturation were compared between mice and humans. A mutual position of CD9/CD81 is shown in human spermatozoa in the acrosomal cap, however in mice, CD9 and CD81 occupy a distinct area. During the acrosome reaction in human sperm, only CD9 is relocated, compared to the relocation of both proteins in mice. The structural modelling of CD9 and CD81 homologous and possibly heterologous network formation was used to propose their lateral Cis as well as Trans interactions within the sperm membrane and during sperm–egg membrane fusion.

## 1. Introduction

CD9 and CD81 are expressed in a large variety of cells [1] and belong to the tetraspanin superfamily (TM4SF), whose members are small (20–50 kDa) proteins [2,3]. Their ability to form homologous partnerships as well as interact heterologously with distinct, non-tetraspanin proteins (including adhesion molecules, receptor and co-receptor molecules, and antigens of major histocompatibility complex or cytoplasmic kinases) represents the key feature of tetraspanins that enables them to create a complex active network on a cell membrane surface called a “tetraspanin web” [4,5,6]. Consequently, tetraspanins are generally viewed as “molecular facilitators” that interact and bring into close proximity specific proteins involved in processes of cell activation and transduction, in particular of cellular development, proliferation, activation, and motility of somatic cells [7]. In a tetraspanin web, very relevant partners of CD9 and CD81 are integrins, mainly β1 integrins. These molecules together create integrin-tetraspanin adhesion complexes and tetraspanins play important role in integrin signalling [8]. In somatic cells, the association of CD9 and CD81 with α3β1 [8,9] and α6β1 integrins was confirmed [10,11,12], and a similar association can be expected in sperm, as the presence and favourable localization of these integrin heterodimers in sperm head was shown by Frolikova et al. [13]. 

Although the majority of up-to-date known molecular interactions mediated by tetraspanins were described within a single cell membrane (*Cis* interaction) [4,14,15], a “partnership” of tetraspanins with proteins in distinct cell membranes (Trans interaction) was also reported [16]. Kovalenko et al. [17] published the existence of both homo- and hetero-dimers of CD9 and CD81. Equally homo-and heterophilic reciprocal interactions of tetraspanins have been described [14,18]. Except tetraspanin-protein interactions, tetraspanin-lipid interactions play a crucial role in tetraspanin web organization and function. 

Therefore, it is highly probable that the proteins expressed on sperm, respectively gametes, are also organized in a tetraspanin web that is active prior and during fertilization, which is supported by the expression of tetraspanin molecules CD9 and CD81 on mouse and bull sperm [19,20]. The crucial role of CD9 and CD81 in fertilization is emphasized by the sterility of CD9/CD81 double null female mice [21]. In contrary the individual CD9 [11,22] or CD81 [21] knock-out female mice displayed reduced fertility when mated with males. On the other hand, both knock-out male mice lacking CD9 or CD81 were fertile [21,22,23]. The published modulation of the possible molecular mechanism of CD81 activity [24] contributed to a better explanation of fertilization events in mammals using a computational approach. Although the proposed model was matched for the CD81 molecule, the cholesterol binding principle can be considered as a key moment for switching between the active and non-active position and therefore binding of a partner protein. Follow-up association of tetraspanins with membrane-curving proteins or lipids, or different tetraspanins with abilities to bind these molecules, could influence the curvature of the cell membrane and formation of various tubular structures (e.g., microvilli) associated with cell adhesion and intercellular communication [25]. The changes of microvilli distribution and their CD9 content were proposed to be responsible for the development of the oocyte membrane block to sperm penetration [26] and female infertility of CD81-deficient mice due to less outward curvature of the oolema was also predicted [25]. The membrane tubular structures formation could be regulated by associations of tetraspanins with actin cytoskeleton via ERM proteins [25].

In this study, the putative mechanism of involvement of CD9 and CD81 in tetraspanin web formation and its activity during sperm preparation for fertilization was addressed with a focus on the species-specific differences between mouse and human. The distribution of CD9 and CD81 molecules in mature sperm and the differences in behaviour of CD81 in the acrosomal exocytosis are described. The structural modelling of CD9 and CD81 homologous and heterologous network formation discussing both lateral Cis interaction within the sperm membrane and Trans interaction during gamete fusion was used to propose the tetraspanin network structure.

## 2. Results

### 2.1. Mutual Position of CD9 and CD81 on Mouse Sperm Head

**Figure 1:**
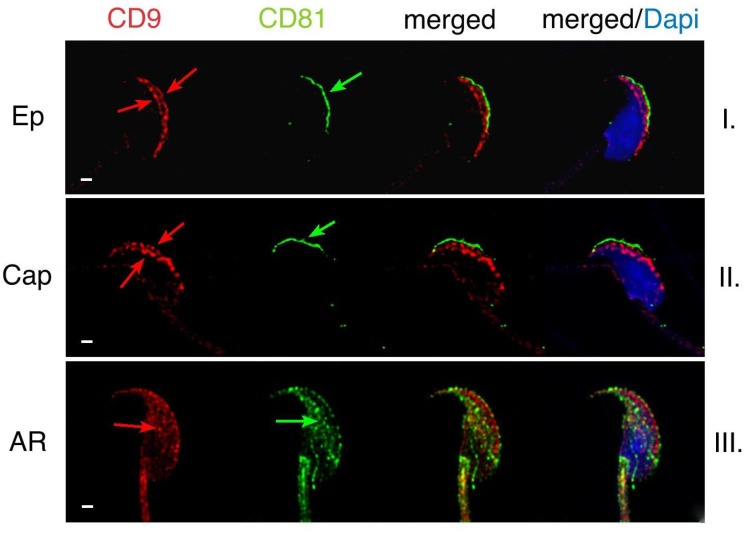
Dynamics of CD9 and CD81 and changes in their mutual localization in mouse sperm captured by confocal microscopy. (**I**) In freshly released epididymal mouse sperm, CD9 (red) is locked over the acrosome vesicle in both of its membrane compartment—inner and outer acrosome membrane (red arrows) and CD81 (green) is present in plasma membrane over the apical acrosome (green arrow). (**II**) During capacitation no changes in localization of both proteins occur, see both red and green arrows. (**III**) After finishing of AR, plasma membrane covering acrosome apical area and outer acrosomal membrane are lost. However, both CD9 and CD81 are still detectable. CD9 and CD81 are relocated in inner acrosomal membrane and in plasma membrane of equatorial segment (red and green arrows). They start in mutual contact. DAPI (blue). Scale bar represents 1 μm.

**Figure 2:**
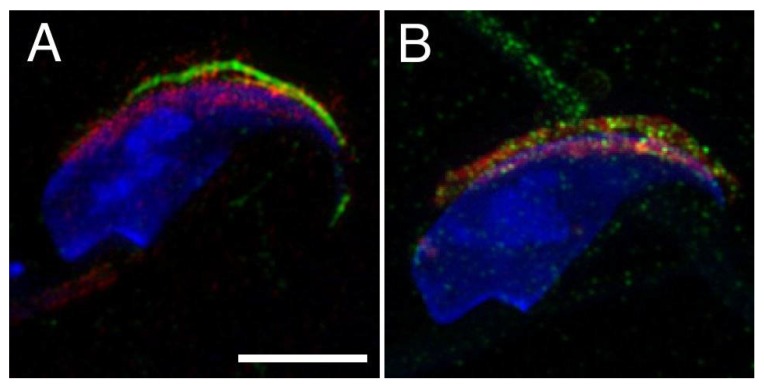
Precise localization of CD9 within the inner and outer acrosomal membranes depicted by structure illumination microscopy (SIM) in mouse epididymal sperm head. (**A**) SIM data confirm localization of CD81 (green) in plasma membrane of apical acrosome area and localization of CD9 in acrosomal membrane and absence of their mutual contact in epididymal sperm; (**B**) CD46 (red) was used as marker for confirmation of presence of CD9 (red) in both outer and inner acrosomal membranes. DAPI (blue). Scale bar represents 5 μm.

To understand the relationship of CD9 and CD81 proteins on sperm and their role in the fertilization process, we investigated their mutual position on mouse sperm head in freshly released epididymal, capacitated and acrosome reacted sperm (Figure 1). The localization of CD81 on plasma membrane covering apical acrosome and CD9 on the acrosomal membrane and relocalization of both tetraspanins into equatorial segment during acrosome reaction (AR) have been already described for each of these proteins individually [19,20]. However, they have never been depicted simultaneously on sperm to address their mutual behaviour. Our results of dual immunofluorescent labelling confirm that CD81 is expressed on the plasma membrane covering the apical acrosome (see green arrow in Figure 1, line I), and CD9 is present on the acrosomal membranes (see red arrows in Figure 1, line I.) in mouse epididymal sperm. Our data showed the presence of CD9 in both outer and inner acrosomal membranes using confocal (see red arrows in Figure 1, line I) and structure illumination microscopy (SIM) (Figure 2). SIM was further used to visualise in detail the accurate dual position of CD9 and CD81. In contrary to CD9, the CD81 protein is localized in the apical plasma membrane covering the acrosomal region in mouse epididymal sperm (Figure 2A). To visualise the localization of CD9, CD46, which is known to be present in both acrosomal membranes, was used as a marker. The localization of CD9 correlates with CD46 using SIM (Figure 2B) and confirms their mutual position. We achieved axial resolution 120 nm for the green channel and 140 for the red channel using the SIM method.

**Figure 3:**
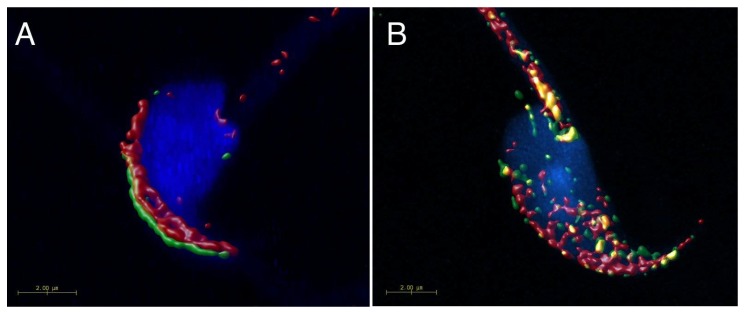
Surface rendering of colocalization map of CD9 and CD81 in mouse epididymal and acrosome reacted sperm. Confocal images and Huygens software were used for better visualisation colocalization area (yellow) of CD9 (red) and CD81 (green). (**A**) No colocalization area was detected on freshly released epididymal sperm contrary to the acrosome reacted sperm (**B**), where the analysis confirmed a mutual colocalization of CD9 and CD81. Colocalization maps are based on Pearson’s correlation coefficient. DAPI (blue) Scale bar represents 2 μm.

The localization of CD9 and CD81 does not change during mouse sperm capacitation (Figure 1, line II), but both proteins relocate to the equatorial segment and partially over the postacrosomal region during AR. Moreover, they start to appear close to each other in the equatorial segment after AR (see arrows in Figure 1, line III). Three-dimensional colocalization maps based on Pearson’s correlation coefficient were created for more accurate visualisation of the colocalization area (Figure 3). No colocalization of CD9 and CD81 was detected in epididymal sperm (Figure 3A), but the areas of mutual colocalization were present in sperm head after AR (Figure 3B).

### 2.2. Mutual Localization of CD9 and CD81 in Human Sperm Head

**Figure 4:**
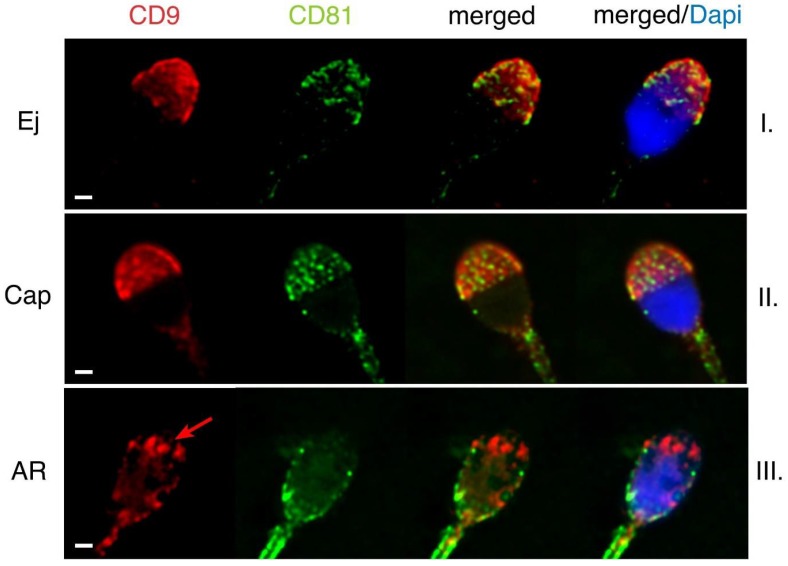
Dynamics of CD9 and CD81 in their mutual localization in human sperm captured by confocal microscopy. (**I**) In ejaculated sperm, both CD9 (red) and CD81 (green) are present in the apical acrosome. (**II**) During capacitation no changes in localization of both proteins occur. (**III**) After completing AR, the plasma membrane covering the apical acrosomal area and the outer acrosomal membrane are both lost. The fluorescent signal of CD9 is located over the equatorial segment (red arrow) and CD81 disappears from the acrosome. After the acrosome reaction, CD81 is mainly detectable in the postacrosomal region of human sperm head. DAPI (blue). Scale bar represents 1 μm.

**Figure 5:**
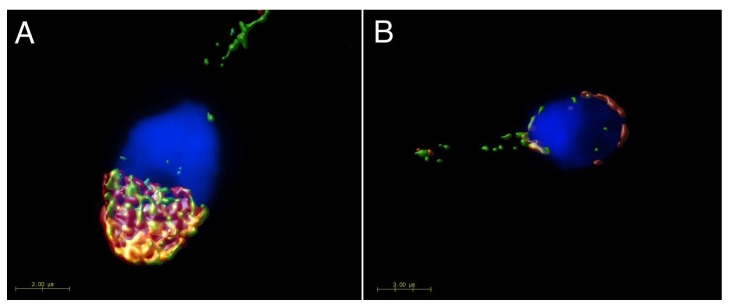
Surface rendering of colocalization map of CD9 and CD81 in human ejaculated and acrosome reacted sperm. Confocal images and Huygens software were used for better visualisation of colocalization area (yellow) for CD9 (red) and CD81 (green). (**A**) Ejaculated human sperm shows a high rate of colocalization of CD9 and CD81 in the acrosomal cap. (**B**) In case of acrosome reacted sperm, CD9 remains to be present in the inner acrosome membrane, however CD81 is hardly detectable across the sperm head. DAPI (blue). Scale bar represents 2 (**A**) and 3 (**B**) μm.

In human ejaculated sperm, the presence of CD9 and CD81 was detected in the apical acrosomal area (Figure 4, line I), and CD81was also partiality present in the post-acrosomal area. In ejaculated human sperm, both proteins are localized within the acrosomal cap but there are differences in the labelling pattern of CD81 and CD9. While CD81 creates very specific non-homogenous dotted pattern, CD9 is evenly distributed (Figure 4). These findings suggest that CD81 could be accumulated in clusters favouring their homophilic interactions. No significant changes in protein localization occurred during capacitation process (Figure 4, line II). When AR is completed, the plasma membrane covering the apical acrosomal area and the outer acrosomal membrane are both lost. The fluorescent signal of CD9 remains in the equatorial segment (see red arrow in Figure 4, line III), but CD81 disappears from the apical area of the acrosomal cap. However, it stays detectable in the postacrosomal region of human sperm head (Figure 4, line III). As in the case of mouse sperm, we created 3D colocalization maps based on Pearson’s correlation coefficient for a more accurate visualisation of the colocalization area and a better interpretation of immunofluorescent results (Figure 5). The ejaculated human sperm show high rate of colocalization (Figure 5A). While nearly no colocalization was detected in acrosome reacted human sperm (Figure 5B).

### 2.3. Immunoprecipitation of CD9 and CD81 Complex from Spermatozoa

**Figure 6:**
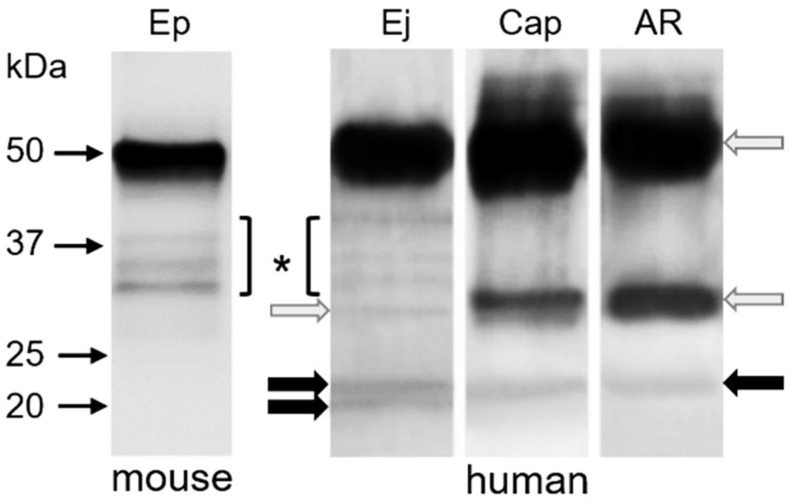
Co-immunoprecipitation of CD9 and CD81 molecules from lysates 5 × 10^7^ of mouse epididymal sperm (Ep), and human sperm (Ej—ejaculated, Cap—capacitated, AR—acrosome-reacted). Detection of CD81 tetraspanin with polyclonal rabbit antibody anti-CD81 (H-121) in CD9 immunoprecipitates using polyclonal rabbit antibody (H-110); grey arrows—heavy and light chains from antibody, black arrows—antibody reaction, * protein bands from rabbit antiserum.

The co-immunoprecipitation experiments on the mouse model did not reveal any mutual interaction between CD9 and CD81 tetraspanin molecules in spermatozoa. Representative figure of detection with CD81 antibody in CD9 epididymal sperm immunoprecipitate did not show a band in expected molecular weight (Figure 6). The co-immunoprecipitation experiments with human spermatozoa proved CD9 and CD81 interactions in different stages of their post-testicular maturation (Figure 6). In CD9 immunoprecipitate from ejaculated and capacitated human sperm, CD81 antibody reacted with two bands in molecular weights of 22 and 24 kDa. On the other hand, the CD81 molecule was detected in CD9 immunoprecipitate from acrosome-reacted sperm only in one protein band of 24 kDa. Strong protein bands showed heavy and light chains of CD9 antibody (Figure 6, grey arrows). 

### 2.4. Determination of Intra-Molecular Disulphide Bonds in CD9 and CD81 Tetraspanins

**Figure 7:**
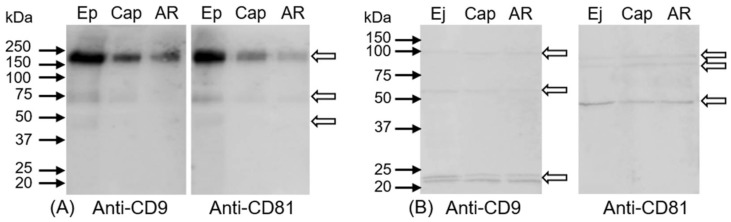
Detection of CD9 and CD81 tetraspanins in mouse (**A**) and human (**B**) sperm extract under non-reducing conditions (5 × 10^6^ sperm cells per lane); Ep—epididymal, Ej—ejaculated, Cap—capacitated, and AR—acrosome-reacted spermatozoa. White arrows indicate antibody reaction. As negative control, blots were incubated with rabbit immunoglobulins. Blots were completely negative.

Western blot analysis under a non-reducing condition was performed for depiction of potential disulphide bonds in tetraspanin molecules. Sperm RIPA lysates from mouse spermatozoa in different post-testicular maturation stages showed high-molecular-weight complexes connected with intramolecular disulphide bridges. Both antibodies, anti-CD9 and anti-CD81, recognized protein band over 160 kDa with decreased intensity from epididymal to acrosome-reacted mouse sperm samples. Furthermore, a protein band of 75 kDa was observed with a lower intensity in capacitated and acrosome-reacted sperm lysates. In epididymal sperm extract, both tetraspanin antibodies recognized diffuse band of 45 kDa (Figure 7A). In all human sperm lysates under non-reducing conditions, the CD9 antibody detected protein bands with molecular weights of 60 and 100 kDa, and a double band in the range of 20 and 25 kDa. The CD81 antibody recognized evident band of approximately 48 kDa and two weaker protein bands between 75 and 100 kDa in all sperm lysates (Figure 7B).

### 2.5. Molecular Modelling

We have obtained an all-atom model of human CD81 by modelling the region not refined by crystallography (residues 38 to 54) using 100 runs of the loopmodel function of MODELLER. The modelling suggests a disordered highly flexible region without strong preference for secondary structure. However, the remaining part of the extracellular domain is stabilized by two disulphide bridges, and the domain is involved in a closed/open conformation change [26].

**Figure 8:**
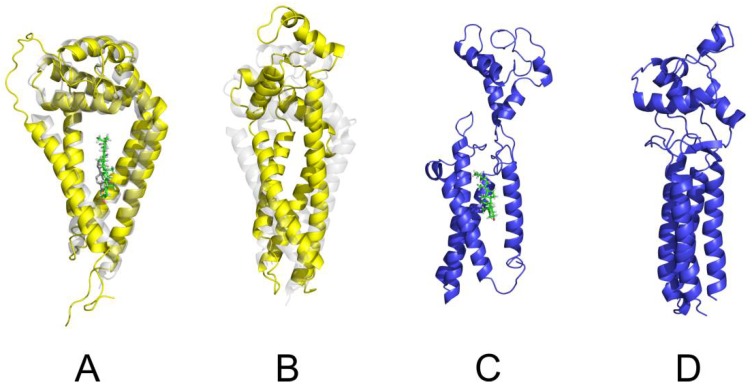
Models of CD81 and CD9 with and without cholesterol. (**A**) CD81 monomer (yellow) with bound cholesterol (green) after 50 ns MD simulation, showing the conical shape of the TM helices. Overlaid is the starting crystal structure (transparent gray); (**B**) CD81 monomer (yellow) without cholesterol after 50 ns MD simulation. The TM helices adopt more compact cylinder like conformation; (**C**) CD9 monomer (blue) binding cholesterol (green) after 50 ns MD simulation. The model suggests that contrary to the CD81 case the extracellular domain is more extended in cholesterol rich environment; (**D**) CD9 monomer (blue) without cholesterol after 50 ns MD simulation showing a tight interaction of the TM helices.

For better understanding of this change, we have performed a series of 50 ns–long MD simulations of the CD81 model in implicit solvation/lipid membrane model; Effective Energy Function 1/Implicit Membrane Model 1 (EEF1/IMM1) and the explicit all atom TIP3P water/DOPC (dioleoyl-phosphatidylcholine) membrane simulations with or without cholesterol present in the binding cavity formed by transmembrane helices (TM). The cholesterol bound CD81 structure indicates (consistently with the crystal structure) a conical shape of the protein with wider separation of TM helices towards the extracellular domain inducing a convex curvature of the membrane bilayer (Figure 8). Within a cholesterol depleted environment, the cavity is not occupied, and TM helices form in a more compact bundle. This potentially leads to a change in membrane curvature which is crucial for sperm-egg fusion at the convex part of the equatorial segment, as the place of first sperm-egg plasma membrane interaction. Without cholesterol binding, the compact arrangement of TM helices would lead to more planar or even a concave membrane bilayer. We have also observed the cholesterol unbinding induced conformational change with the extracellular domain opening as a lid. The newly accessible residues, including the disordered region, probably play a role in recognition by a so far unknown binding partner and could facilitate both Cis and Trans interactions.

**Figure 9:**
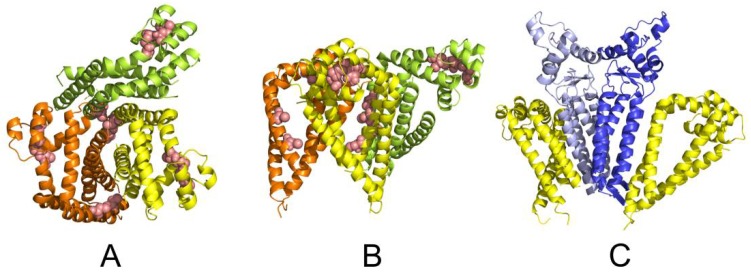
A summary of oligomer forming interfaces of CD81 and CD9. (**A**,**B**) Top and side view showing two possible binding modes of CD81 monomer (yellow) as predicted by flexible side-chain docking. The yellow-orange CD81 dimer displays a strong non-covalent interaction, while the less stable yellow-lime CD81 dimer can be further stabilized by formation of an intermolecular disulphide bridge. The Cys residues are shown in pink. A rotation of the covalently bound lime CD81 moiety could recruit another CD81 subunit leading to stepwise formation of CD81 oligomer stretch (see Figure 10); (**C**) The CD9 dimer (shades of blue) displaying two potential binding modes with CD81 moiety (yellow) as predicted by flexible side-chain docking.

Further, we have performed a series of protein–protein flexible side-chain docking using ClusPro. Two principal CD81 binding modes were identified (Figure 9A,B). While the most probable dimer interface shows the TM Cys residues pointing in opposite direction and the dimer is stabilized only non-covalently, the second dimer interface allows further covalent stabilization by forming a disulphide bridge between neighbouring Cys residues across monomers. The formation of a disulphide bridge between CD81 monomers is also supported by our experimental results.

An all-atom model of the human CD9 protein was obtained using the I-TASSER suite of programs. We have performed 50 ns long MD simulations of the CD9 model with or without cholesterol present. The modelling suggests that the effect of possible cholesterol binding to CD9 is not as profound as in CD81 case. The results show that the TM helices of CD9 are rather compact and arranged almost parallel to each other in both cases. The extracellular domain is only weakly sensitive to cholesterol binding and remains in the open conformation. This may be interpreted that CD9 takes less active or no role in shaping membrane curvature contrary to CD81. 

In order to identify a possible CD9 dimer, we performed the protein-protein flexible side-chain docking using ClusPro. The results suggested a dimer with the TM helices of CD9 monomers in a V shape arrangement (Figure 9C). The docking followed by a 50 ns MD simulation of the CD9 dimer in implicit solvent/membrane environment suggests that the extracellular domain is involved in CD9 dimer formation employing the short helix (residues 138 to 151) as dimerization interface. The outward facing surface can serve as an interface for other binding partners.

No disulphide bridges involving Cys residues within the transmembrane region of CD9 dimer were suggested by the docking. This is consistent with the Uniprot annotation for the transmembrane Cys residues which are expected to be post translationally modified by palmitoylation, probably utilized more in CD9 web stabilization during sperm maturation and acrosome reaction protein dynamics than in cholesterol binding. Moreover, the palmitoylation is beneficial during membrane fusion when could ease the CD9 transfer across the membranes. CD9, in contrary to CD81, also exhibits more extended extracellular domain in dimer (Figure 9C) which would be recipient towards the trans interactions. On the other hand, the CD81 does not show considerable palmitoylation according the Uniprot, and as a result would allow binding of cholesterol or phospoholipides. Our experimental data also showed no oligomers stabilized by intermolecular disulphide bridges for CD9. However, similarly to the CD81, two intramolecular disulphide bridges stabilizing the extracellular domain of CD9 are expected to form based on the sequence homology. 

## 3. Discussion

Events prior and during sperm–egg fusion are connected with drastic membrane reorganization of both gametes [27]. One of the “regulators” of these processes is cholesterol efflux. Besides the membrane microdomains enriched in cholesterol defined as rafts, other membrane “structures” called tetraspanin-enriched microdomains (TEM) are presented within the membrane [5,28], and tetraspanin–tetraspanin interactions are also regulated by lipids, including the palmitate moieties that are attached to tetraspanins, membrane cholesterol, and gangliosides [28,29,30,31,32]. Based on recently detected open conformation (cholesterol free) of CD81 tetraspanin, the regulation of subcellular localisation of their partner proteins in response to differences on cholesterol concentration could be proposed, via the ability to tightly bind partner proteins [24]. This feature of CD81 is likely to be also utilized by mammalian sperm during their maturation call capacitation when major membrane rearrangement including cholesterol efflux takes place [33]. In vitro capacitation disrupts lipid raft domains and causes a shift in the overall membrane fluidity in the plasma membrane [34], which may induce the interaction of raft resident proteins to initiate signalling pathways associated with the capacitation [34]. Based on the data of Hogue et al. [35] the interaction of proteins from rafts with tetraspanins within TEM could be expected. Within mammalian sperm lipid rafts, several molecules have been documented with affinity for the ZP and egg plasma membrane including sperm fusogenic protein IZUMO1 [34], and according to Tanphaichitr et al. [36], the sperm lipid rafts are platforms of ZP binding molecules on the sperm plasma membrane. Beside others, capacitation-associated changes in sperm lipid rafts play a role in positioning IZUMO1 into the equatorial fusogenic region during AR [37,38]. The cooperation with rafts also seems to be probable for CD9 and CD81 in mice. These tetraspanins share location and dynamic rearrangement during AR and they are part of the multiprotein network participating during membrane fusion. The questions have remained to be answer whether CD9 and CD81 crosslink into a uniform heterologous network, and if so, whether these two tetraspanins utilise covalent or non-covalent bonds. Moreover, are these predicted interaction species specific or could they also depend on the maturation stage of the sperm? Our results from immunofluorescent staining and consequently from co-precipitation suggest that both are probably true. We showed crucial differences in localization of CD9 and CD81 between human and mouse sperm and between individual maturation stage of sperm. Our results from co-precipitation experiments showed potential mutual interaction of CD9 and CD81 tetraspanins in human spermatozoa which coincide with results of immunofluorescent staining that confirm mutual colocalization of studied proteins in acrosomal cap area. Consequently, in epididymal mouse sperm, where we noticed completely different localization of CD9 and CD81, no complexes of these proteins were detected by co-precipitation. However, contrary to Ito et al. [19] that described CD9 as an inner acrosomal membrane associated protein, our data showed the presence of CD9 in both outer and inner acrosomal membranes. Moreover, in human ejaculated sperm CD9 immunoprecipitates, the CD81 antibody recognized two bands of 22 and 24 kDa. Antibody signal of lower protein band decreased during capacitation and was completely absent in acrosome-reacted sperm. This may be caused by leaving of antibody epitope from one of CD81 isoforms or number of spermatozoa undergoing spontaneous acrosome reaction, where isoform with lower molecular weight disappeared together with acrosomal cap. 

For disulphide bridges modelling, lysates of human spermatozoa were prepared in non-reducing conditions. CD81 molecule forms dimmers connect with S-S bonds. This presumptive CD81 dimmer has been already described by Stipp et al. [39]. On the other hand, CD9 protein was found predominantly as a monomer (double band between 20 and 25 kDa) without disulphide bridges. Additionally, some covalent complexes were detected in approximate molecular weight of 60 kDa. Moreover, covalent complexes in human sperm lysates differ in CD9 and CD81 detection. However, according to co-precipitation experiments, these two tetraspanin molecules may interact non-covalently. Nevertheless, CD9 and CD81 molecules might be covalently linked with other tetraspanins or protein molecules. In the structure of the CD9 molecule, there are 10 cysteins and six are palmitoylated. Four of them are located in extracellular domain without palmitoylation and covalent interaction with other proteins may be formed more likely in extracellular domain. In samples of mouse spermatozoa, both antibodies detected high-molecular-weight complexes (over 160 kDa), presumably octamers. Although detection by both antibodies showed bands in the same molecular weight, co-precipitation experiments revealed that these complexes are not covalently formed by CD9 and CD81. However, as mentioned above also these complexes are likely formed by CD9 and CD81 molecules with other proteins, such as ERMs [40] and integrins, CD19, CD21, and CD45 [41]. Interestingly, there is evident decrease of signal intensity of antibodies reaction in capacitated and acrosome reacted sperm lysates, probably due to spontaneous and induced acrosome reaction [42], respectively.

**Figure 10:**
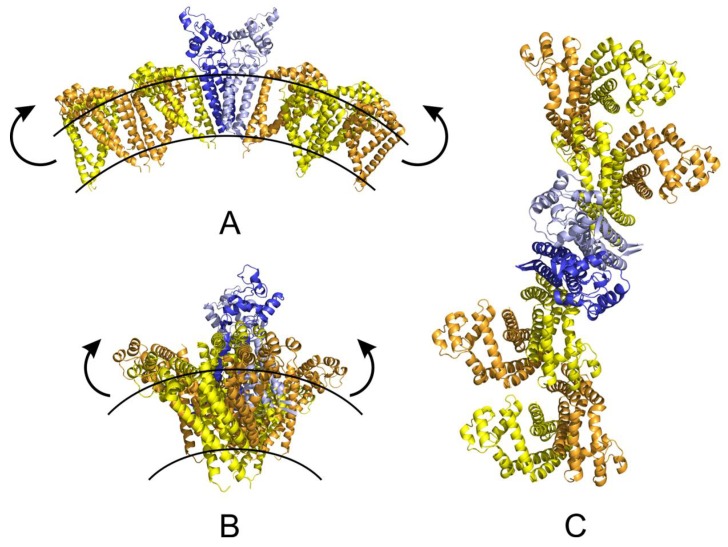
The proposed arrangement of the CD81/CD9 tetraspanin network. A complex CD9 and CD81 network can be formed combining the extended CD81 oligomer formed by alternating non-covalent (yellow-orange) and disulphide bridge stabilized dimers (orange-orange). A dimer of dimers is depicted for simplicity, however, longer CD81 oligomer stretches could be formed. A CD9 non-covalent dimer (shades of blue) can then interact with two such oligomers. (**A**,**B**) Side and front view along the CD81 oligomer stretch (a membrane—black lines—would be in horizontal orientation) showing the possible CD81/CD9 network (**C**) Top view (membrane in plane). Under low cholesterol conditions the dissociation of CD81 bound cholesterol might lead to more compact TM region within CD81 stretches changing its conical to cylindrical conformation which would result in lower area of the “upper” leaf of the bilayer and bending of the membrane (indicated with arrows in (**A**,**B**)).

**Figure 11:**
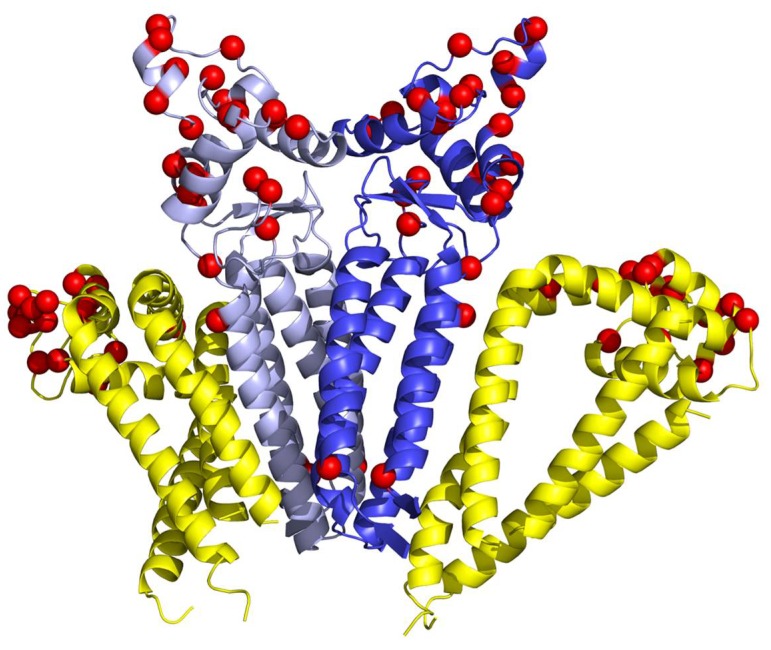
The position of non-identical amino acids between human and mouse CD81 and CD9 projected onto the model of CD9/CD81 complex (Figure 9C). The differences in amino acid sequence are depicted as red spheres. The CD81 moiety (yellow) contains 17 differences in the extracellular domain, while the CD9 (shades of blue) differs in 25 amino acids located also at the CD9/CD81 interface. These differences could be responsible for species specific interaction between CD9 and CD81 as well as recognition by other proteins.

To suggest a potential structure and function of a tetraspanin web involving CD81 and CD9 moieties, we performed also a series of docking and MD simulations with different CD81/CD9 stoichiometry. Based on the results of all CD9 and CD81 simulations we can speculate that a complex network can be formed combining the extended CD81 oligomer formed by alternating non-covalent and disulphide bridge stabilized dimers. A CD9 non-covalent dimer can then interact with two such oligomers. Moreover, tetraspanins via their ability to associate with membrane-curving proteins or lipids, or different tetraspanins may influence the curvature of the membrane and formation of various tubular structures [25] participating in formation of fusogenic domains. The CD9 and CD81 combined network can be significantly influenced by the presence or absence of cholesterol in the lipid bilayer leading to changes in membrane curvature. The proposed arrangement of the CD81/CD9 tetraspanin web is summarized in Figure 10. Under low cholesterol conditions the dissociation of CD81 bound cholesterol might lead to more compact TM region within CD81 stretches changing its conical to cylindrical conformation which would result in lower area of the “upper” leaf of the bilayer and bending of the membrane. This CD81 ability may be suspected to play a crucial role for facilitating sperm-egg plasma membrane interaction and fusion as convex part of the equatorial segment servers for primary recognition and perpendicular attachment of mammalian sperm to the oolema. The modelling was dealing with human variants of CD9 and CD81 proteins. The analysis of homology between mouse and human variants revealed that the transmembrane domain residues are mostly sequentially conserved. However, the extracellular domains differ significantly as summarized in Figure 11.

The responsibility of CD9 tetraspanin for strong adhesion generating fusion competent sites between sperm and egg plasma membrane has been shown [15] as well as the importance of cholesterol as a key mediator of membrane curvature during fusion events [43,44]. A complex CD9 and CD81 network can be formed combining the CD81 oligomer utilizing Cis interaction both non-covalent and disulphide bridge which lead to the complex stabilization. A CD9 non-covalent dimer can then interact with two such CD81 oligomers. Buschiazzo et al. [44] assumed that cholesterol efflux during the sperm capacitation make sperm membrane more fluid and able to undergo greater positive curvature to adapt a more ordered oocyte membrane at the moment of fusion. Specifically, under low cholesterol conditions, the dissociation of CD81 bound cholesterol might lead to more compact transmembrane region within CD81 stretches changing its conical to cylindrical conformation. This feature would consequently result in bending of the bilayer membrane. Taken together experimental results and structural modelling, we proposed, that due to the ability of the CD81 molecule to bind and release cholesterol accompanied by stable covalent formation of the network, CD81 might be considered as a regulator of dynamic machinery within the tetraspanin web on the sperm plasma membrane. On the other hand, CD9 in weak interaction with CD81 might be more likely involved in tetraspanins web stabilization and play a role in tetraspanins facilitated trans interaction at sperm–egg membrane recognition by employing the more extended extracellular domain.

## 4. Materials and Methods

### 4.1. Primary Antibodies

In our experiments, the following primary antibodies were used: polyclonal rabbit anti-CD9: H-110, sc-9148, raised against a peptide mapping within an internal region of CD9 (101–210 AA) of human origin; polyclonal rabbit anti-CD81: H-121, sc-9158, raised against a peptide mapping within an internal region (90–210 AA) of CD81 of human origin; polyclonal goat anti-CD81: Q-14, sc-31234, raised against a peptide mapping within an N-terminal extracellular domain of CD81 of human origin. All these antibodies were produced by Santa Cruz Biotechnology, Santa Cruz, CA, USA. Anti-CD46 (HM-1118; Hycult Biotech, Uden, The Netherlands) was used as a marker of acrosomal membrane.

### 4.2. Animals

C57BL/6J mice were used for the experiments. They were purchased from the Animal Resources Centre or produced by the animal breeding facilities of the Institute of Biotechnology. The mice were housed in the IMG animal facilities, Institute of Molecular Genetics of Czech Academy of Science, Prague, and food and water were supplied ad libitum. The mice were healthy 10–12 weeks old five animals with no sign of stress or discomfort. All animal procedures and experimental protocols were approved by the Animal Welfare Committee of the Czech Academy of Sciences (Animal Ethics Number 66866/2015-MZE-17214, 18 December 2015). 

### 4.3. Human Samples

Human ejaculates were obtained from IVF Center Gennet (Prague, Czech Republic) with the informed consent of healthy donors and in accordance with the Institutes’ Human Ethics Committee guidelines. Spermiograms were evaluated in andrological laboratory at IVF Center. Only normospermic samples according to WHO laboratory manual (2010), five different healthy donors, were used in our study. After liquefaction, 1 mL of ejaculate was subjected to discontinuous centrifugation gradient (55%/80%) SupraSperm System (Origio, Måløv, Danmark) and centrifuged at 300 *g* for 20 min at room temperature.

### 4.4. Capacitation and Acrosome Reaction

#### 4.4.1. Mouse Sperm

In this study, 35 mm Petri dishes obtained from Corning (New York, NY, USA) were used for capacitation in vitro. Spermatozoa, which were recovered from the distal region of *cauda epididymidis*, were placed in capacitating M2 medium (Sigma-Aldrich, Prague, Czech Republic) and left in an incubator for 10 min at 37 °C under 5% CO_2_ to relax sperm. After that, the concentration of stock sperm in medium was adjusted to 5 × 10^6^ sperm/mL in 100 μL of M2 medium under paraffin oil and left to capacitate for 90 min. Calcium Ionophore (CaI, A 23187, Sigma-Aldrich), at a final concentration of 5 μM was added to the capacitated sperm and acrosome reaction was induced for an additional 90 m. The viability and motility of the sperm population was checked throughout the whole experiment.

#### 4.4.2. Human Sperm

Purified human spermatozoa of 5 × 10^6^ cells were capacitated in 0.5 mL of SpermPreparation Medium (Origio, Måløv, Danmark) for 2 h at 37 °C under 5% CO_2_. Acrosome reaction was induced by calcium ionophore A23187 (Sigma-Aldrich) in final concentration of 10 µM for 1 h at 37 °C under 5% CO_2_. Acrosome-reacted sperm were evaluated on the absence of PNA-lectin labelling (VectorLaboratories, Burlingame, CA, USA) under the fluorescent microscope.

### 4.5. Dual Immunofluorescent Staining of CD81 and CD9

Ejaculated or epididymal, capacitated, and acrosome-reacted sperm were used for preparation of cells smears. Sperm were washed twice in tube with PBS, smeared onto glass slides, and air-dried. Sperm smears were fixed with 3.7% formaldehyde in PBS (pH 7.34) at room temperature for 10 min, followed by washing in PBS. Sperm were blocked with 5% BSA in PBS for 45 min and incubated with primary antibodies: goat polyclonal IgG anti-CD81 (Q14) diluted 1:20 in PBS and rabbit polyclonal IgG anti-CD9 (H110) diluted 1:20 in PBS over night at 4 °C, followed by 1 h of incubation with both secondary antibody together: Alexa Fluor 568 donkey anti-rabbit IgG (H + L) or Alexa Fluor 488 donkey anti-goat IgG (H + L) (Life Technologies, Prague, Czech Republic) diluted 1:500 in PBS at room temperature. After washing, the slides were mounted into a Vectashield mounting medium with DAPI (Vector Lab., Burlingame, CA, USA). Fluorescent images were collected with high-end confocal microscope Carl Zeiss LSM 880 NLO at Imaging Methods Core Facility at BIOCEV (Vestec, Czech Republic). Huygens Professional version 17.10 (Scientific Volume Imaging, Hilversum, The Netherlands, Available online: http://svi.nl) software was used for deconvolution of confocal images and for colocalization maps. An open-source software Fiji [45] was used for image processing. 

### 4.6. Super-Resolution Microscopy

Freshly released epididymal sperm were used for SIM super-resolution microscopy. Sperm were collected, as described previously, with the following differences. Sperm samples were always prepared onto high precision cover glasses (thickness No. 1.5 H, 170 ± 5 μm, Marienfeld, Germany). Moreover, after the application of the primary and secondary antibodies, sperm were incubated for 5 min with DAPI (0.85 μg/mL, Thermo Scientific, Waltham, MA, USA) and washed 3× in PBS. At the end, sperm were washed 1× in distilled water and air-dried. Dry samples were covered with 90% glycerol with 5% anti-fade N-propyl gallate (Sigma-Aldrich). Multi-colour SIM super-resolution images were obtained by Zeiss Elyra PS.1 inverted microscope at Laboratory of confocal and fluorescent microscopy of Faculty of Science (Charles University, Prague, Czech Republic). An open source software Fiji was used for another image processing.

### 4.7. Protein Extraction

Sperm suspension (mouse epididymal, human ejaculated, mouse and human capacitated and acrosome-reacted) were collected, washed in PBS and pellets of 5 × 10^7^ sperm cells were lysed in 100 µl of RIPA buffer (150 Mm NaCl; 1% NP-40; 0.5% sodium deoxycholate; 0.1% SDS—Sodium Dodecyl Sulphate; 50 mM Tris pH 7.5 and protease inhibitors) for membrane protein extraction. Two-times concentrated non-reducing sample buffer with 8 M urea were added in 1:1 (*v*/*v*) ratio and vortexed for 2 min at RT.

### 4.8. Co-Immunoprecipitation of CD9 and CD81 Molecules

Sperm suspension (5 × 10^7^) was lysed in 100 µL of 1% CHAPS at 4°C for 1 h. Lysates were centrifuged at 20.000 *g* for 15 min at 4 °C. Then supernatant was incubated with polyclonal antibody anti-CD9 (H-110) in final amount 5 µg per sample for 2 h at 4 °C in rotator. Twenty µL of washed Dynabeads™ Protein G (Invitrogen, Carlsbad, CA, USA) were added and incubated for 1 h at 4 °C in rotator. Precipitates bound to Dynabeads were separated in magnetic rack (Invitrogen) and washed with RIPA for 5 min at 4 °C in rotator. This procedure was repeated for three times. Co-immunoprecipitated complexes were eluted from Dynabeads by incubation in reducing sample buffer for 5 min at 100 °C.

### 4.9. SDS-PAGE with Immunoblotting

SDS-electrophoresis and immunoblotting technique was used for CD9 and CD81 detection and was carried out using protocols based on standard methods [46,47]. Samples containing protein equivalent to 5 × 10^6^ sperm cells were run on a 4% stacking and 12% running SDS polyacrylamide gel using Precision Plus Protein™ Dual Color Standards (Bio-Rad, Hercules, CA, USA) as molecular weight markers. After transferring proteins onto a PVDF (Polyvinylidine Fluoride) membrane, nonspecific sites were blocked with SuperBlock (PBS) Blocking Buffer (Thermo Scientific). CD9 and CD81 molecules were identified by the primary polyclonal rabbit antibodies anti-CD9 (H-110) and anti-CD81 (H-121) diluted 1:500 in PBS, followed by a peroxidase goat anti-rabbit IgG secondary antibody (Bio-Rad) diluted 1:3000. Antibody reaction was visualised by chemiluminescent substrate Super Signal West Pico (Pierce, Rockford, IL, USA). As a negative control, blots were incubated with immunoglobulins from rabbit serum (Sigma-Aldrich) in the same concentration as primary antibody.

### 4.10. Molecular Modelling of CD9 and CD81 Tetraspanin Web

The amino acid sequences of studied human proteins were obtained from the UniProt database [48] as deposited under following entry names: CD9 (P21926) and CD81 (P60033). The all atom model of the CD81 was built with MODELLER [49] version 9.14 using the CD81 crystal structure (5tcx) [24] as template. The residues missing in the template were refined using the loopmodel function. The I-TASSER standalone package version 5.0 [50] was used to obtain homology models of CD9. The prediction of protein-protein interactions employed the locally available version of the ClusPro 2.0 protein-protein docking server [51]. The parameters of implicit solvation/lipid membrane model (EEF1/IMM1) [52] as well as the all atom DOPC membrane simulations were assigned using the web-based graphical user interface CHARMM-GUI (charmm-gui.org) [53]. The molecular dynamics simulation of suggested complexes was performed using the CHARMM [54] version c41b1 molecular dynamics package. The molecular graphics was prepared using Pymol [55] version 1.8.4.

### 4.11. Data Analysis

Huygens Professional version 17.10 (Scientific Volume Imaging, Hilversum, The Netherlands, Available online: http://svi.nl) was used for visualisation mutual position of individual proteins based on surface rendering of the colocalization analysis. A colocalization analyser computed a Pearson’s correlation coefficient and created a 3D colocalization map. The Pearson’s correlation coefficient expresses the rate of correlation of colocalizing channels in a dual-colour image and gives a value between minus 1 to plus 1. In this case, 1 means an absolutely positive correlation, 0 means no correlation and −1 means a perfect anti-correlation. The value between 0.5 and 1 is interpreted as colocalization. Costes method was used for a background estimation.
